# Temperature-controlled thermophilic bacterial communities in hot springs of western Sichuan, China

**DOI:** 10.1186/s12866-018-1271-z

**Published:** 2018-10-17

**Authors:** Jie Tang, Yuanmei Liang, Dong Jiang, Liheng Li, Yifan Luo, Md. Mahfuzur R. Shah, Maurycy Daroch

**Affiliations:** 1 School of Pharmacy and Bioengineering, Chengdu University, Chengdu, 610106 China; 2 School of Environment and Energy, Peking University Shenzhen Graduate School, Shenzhen, 518055 China

**Keywords:** Biodiversity, Hot springs, Thermophile, MiSeq platform, 16S rRNA, Ganzi

## Abstract

**Background:**

Ganzi Prefecture in Western China is situated geographically at the transition regions between Tibetan Plateau and Sichuan Basin in a highly tectonically active boundary area between the India and Eurasia plates. The region hosts various hot springs that span a wide range of temperature from 30 to 98 °C and are located at high altitude (up to 4200 m above sea level) in the region of large geothermal anomalies and active Xianshuihe slip-fault that has been active since Holocene. The site represents a biodiversity reservoir for thermophiles, yet their diversity and relationship to geochemical parameters are largely unknown. In the present work, bacterial diversity and community structure in 14 hot springs of Ganzi were investigated using Illumina MiSeq sequencing.

**Results:**

Bacterial community compositions were evidently distinct among the 14 hot springs, and the bacterial communities in hot springs were majorly abundant in phyla *Aquificae*, *Cyanobacteria* and *Proteobacteria*. Both clustering and PCoA analysis suggested the existence of four bacterial community patterns in these hot springs. Temperature contributed to shaping bacterial community structure of hot springs as revealed by correlation analysis. Abundant unassigned-genus sequences detected in this study strongly implied the presence of novel genera or genetic resources in these hot springs.

**Conclusion:**

The diversity of hot springs of Ganzi prefecture in Western Sichuan, China is evidently shaped by temperature. Interestingly disproportionally abundant unassigned-genus sequences detected in this study show indicate potential of novel genera or phylotypes. We hypothesize that frequent earthquakes and rapidly changing environment might have contributed to evolution of these potentially new lineages. Overall, this study provided first insight into the bacterial diversity of hot springs located in Western Sichuan, China and its comparison with other similar communities worldwide.

**Electronic supplementary material:**

The online version of this article (10.1186/s12866-018-1271-z) contains supplementary material, which is available to authorized users.

## Background

Microbial populations in terrestrial hot springs have been a subject of interests and concerns of microbiologists. The hot springs around the world have been extensively studied, particularly in Yellowstone National Park (YNP), USA [1], Japan [2], United States Great Basin [3], Iceland [4], and Malaysia [5]. These sites possess diverse physicochemical properties and biological species. In China, similar studies also have been conducted on hot springs, primarily at Tengchong County, Yunnan Province [6, 7] and Tibetan Plateau [8]. Many springs at Tengchong harbored similar microbial communities at the phylum and family/genus levels, as compared to springs at Yellowstone National Park, probably due to the fact that both were volcanically driven with acidic springs [6]. Diverse microorganisms were also documented in Tibetan hot springs, including archaeal phyla *Crenarchaeota*, *Euryarchaeota*, and *Thaumarchaeota*, and various bacterial phyla such as *Cyanobacteria*, *Chloroflexi*, *Chlorobi*, *Proteobacteria*, and *Firmicutes* [8]. Although similarities could be found between different geographical fields, the microbial composition usually differs due to the great impact of physicochemical conditions, such as temperature and pH, on microbial diversity and distribution [3, 6]. Therefore, unique microbial communities are often shaped by distinct niches.

Ganzi Prefecture is located in Western Sichuan Province of China and geographically at the transition regions between Tibetan Plateau and Sichuan Basin. Tectonically, Ganzi is situated at the collision boundary between the India and Eurasia plates [9]. Active new tectonic movement resulted in the abundant hot springs throughout the Ganzi prefecture [9]. The hot springs in Ganzi span a wide range of temperature from 30 to 98 °C and are located at high altitude (up to 4200 m above sea level) [10] in the region of large geothermal anomalies and Xianshuihe slip-fault that has been active since Holocene [11]. In last 300 years four large earthquakes exceeding magnitude 7 occurred in Ganzi prefecture along Xianshuihe fault. Smaller earthquakes occur with large frequency every several years with most recent earthquakes in 22nd of November 2015 (Kangding earthquake, magnitude 6.3), 11th of May 2016 (Luhuo earthquake, magnitude 3.9), and 23rd of September 2016 (Litang earthquake, magnitude 5.1) [11]. These earthquakes result in significant alterations of hot spring ecosystems as they very often bring significant changes of water flow patterns and temperature shifts, disappearance of old and emergence of new hot springs. For example, it has been reported that in the aftermath of Songpan-Pingwu earthquake of 1976 (magnitude 7.8) water temperature of Yulingong spring increased from 89 to 94 °C, an opposite trend was reported for many springs after Kangding Zhetuotang earthquake of 1950 (magnitude 7.8) [11]. Presumably many more similar changes were not reported. This makes the site a very interesting place for the study as dramatic events like earthquakes can completely reshape the environment and result in fast emergence of new phenotypes or even species [12]. Probably the only well documented example of how dramatic changes to the environment brought by an earthquake result in accelerated evolution comes from the study of sticklebacks at the Prince William Sound and the Gulf of Alaska [13]. On 27 March 1964, magnitude 9.2 earthquake elevated islands of the sound by 3.4 m creating freshwater ponds from marine habitat. As a result of an extreme event the resident fish populations have changed dramatically and new phenotypes have been observed within few decades from the event. SNP analysis suggests that extent of change in genomes was striking. After only 50 generations genomes of newly evolved fish diverged from their marine ancestors as much as some older post-glacial freshwater stickleback did for 13,000 years [13]. Hot springs of Ganzi are being reshaped by earthquakes with very high frequency (see Additional file 1: Table S1) which makes them an excellent site for search of phylogenetically and functionally distinct microbial communities. To date such studies were lacking. It will be therefore interesting to look into the biodiversity of thermophiles in Ganzi and in this first study to compare their bacterial diversity and community structure between different springs and with other geothermal environments around the world. Particularly interesting aspects of these springs are presumed historical changes of temperature, water composition and local geological conditions that were shaped by frequent, recent earthquakes. We are interested if these conditions form a strong evolutionary pressure for quick diversification of bacterial species and potentially accelerated evolution of new species and phylotypes.

Numerous approaches and techniques have been developed for exploring the bacterial diversity in hot springs. Basically, these methods were classified into two categories, namely culture-dependent and culture-independent techniques. The culture-dependent approaches were traditionally used and valuable for isolating strains and further exploring their properties [14, 15]. The isolation and characterization of thermophiles is of practical importance because these isolates may have biotechnological potential with regard to the production of useful biomolecules [16]. Our group has recently performed a culture-dependent study of these sites with a focus on cyanobacterial diversity and identified five potential new phylotypes of thermophilic cyanobacteria belonging to the family Leptolyngbyaceae [17]. Disadvantage, however, is evident for this technique, in that the majority of microorganisms are unculturable and only certain classes of microorganisms can be targeted with a single experiment. In light of limited information of microbial diversity provided by culture-dependent method, culture-independent techniques, primarily molecular methods, have been employed to phylogenetically characterize the microbial diversity in hot springs, and were proven to provide a more comprehensive assessment of microbial populations [18]. These approaches include direct 16S rRNA gene amplification and cloning, differentiation using amplified fragment length polymorphism (AFLP) [19], amplified ribosomal DNA restriction analysis (ARDRA) [20], denaturing gradient gel electrophoresis (DGGE) [21], and random amplified polymorphic DNA (RAPD) [22]. Numerous combined approaches have been also developed for the study of microbial communities, including scanning electron microscopy (SEM) combined with community level physiological profiles (CLPPs) [23] and other PCR/sequencing-based techniques [24, 25].

In the last decade, next-generation sequencing (NGS) emerged as a powerful tool for elucidating the biodiversity of resident microbiota from diverse environmental samples. This approach is based on millions of partial 16S sequences amplified from metagenome, which alternatively was known as targeted or amplicon metagenomics. Among the sequencing platforms, 454 was the most commonly used sequencing platform for 16S rRNA gene sequencing, due to a read-length advantage over alternative platforms at the time of its release [26]. However, more recently there is increasing interest in using IonTorrent, PacBio, and Illumina platforms, as driven by researchers’ customized demand on sequence quality, the number of reads generated, and the length of the sequences [27]. Recently, MiSeq, Illumina-based strategy, was reported to have great potential in 16S rRNA gene sequence studies, because it generates longer sequence reads than previous releases of this technology, and its performance and cost are tractable to the needs of individual investigators [28]. Besides, the Illumina-based strategies are able to generate the largest amount of sequence data per unit cost, and MiSeq was capable of generating 8.5 Gbp using paired 250-nt reads (i.e., 17 million pairs of reads) in a single run [27]. Therefore, MiSeq platform is a feasible and robust approach to characterize bacterial communities in hot springs.

As mentioned above, a comprehensive census of the bacterial diversity is still lacking for the hot springs in western Sichuan. In the current study, the V4-V5 hypervariable regions of prokaryotic 16S rRNA genes were amplified from the metagenome of hot spring samples. The primary objective of present study was to census high temperature springs in western Sichuan and to investigate the bacterial community composition and diversity in the hot springs. The relationships between biodiversity and physicochemical conditions (pH temperature and spring altitude) were also analyzed. This study provided a first insight into the bacterial composition in hot springs of western Sichuan and expanded the current understanding of these niches.

## Methods

### Sample collection

Samples from sites described in Table 1 were collected on 15th to 17th of May 2016 from hot spring: mats, sinters, sediments and spring water. Mats, sinter, and sediment samples were directly transferred into sterile 50 mL DNAse/RNAse/pyrogen-free Falcon tubes and stored in dark in a mobile battery-operated cooling unit at <8 °C. Water samples (at least 3 L) containing microorganisms, were concentrated using 0.2 μm membrane (Whatman) and a portable battery-operated pump on site before being stored at same conditions. Water was collected evenly from entire volume of the spring. Mats, sediments, and sinters were collected from multiple points within a spring and mixed together to ensure uniformity. The study followed institutional and national guidelines. No specific permits were required for sampling, none of the locations were in protected, endangered or privately-owned site.

**Table 1:** Information summary of sampling sites

Sample No.	Spring name (region)	pH	Temperature (°C)	Altitude (m)	GPS location	Description
ED	Erdaoqiao	6.32	40.8	2600	30°05′14″ N 101°56′55″ E	Constructed rectangular pool with length 100–110 cm, width ~ 60 cm, and depth ~ 35 cm. The pool is paved with tiles and connected to the spring outflow. Clear water and sulfur smell. Green mats on the edge of pond. Sample type includes mats and water.
ZG-1	Zhonggu village	6.35	53.1	3200	30°15′57″ N 101°52′24″ E	Walled rectangular cement pool with length ~ 500 cm, width ~ 320 cm and depth ~ 100 cm. Five outflows at the bottom of the pool. Green mats on entire bottom of the pond. A pipe outlet. Sample type includes mats and water.
ZG-2	Zhonggu village	6.62	55.5	3200	3 m away from ZG-1	Shallow small natural circular mud pool with slow outflow. Roughly rounded with diameter ~ 60 cm, depth ~ 10 cm. Clear water. Green mats on the edge of pond. Sample type includes mats, sediment and water.
ZG-3	Zhonggu village	7.41	63.7	3200	50 m away from ZG-1	Round natural pool with diameter ~ 150 cm, depth 45 cm. Slow outflow at the bottom of the pool. Floating mats. Sample type includes mats and water.
ZG-4	Zhonggu village	8.5	85	3200	500 m away from ZG-1	Shallow round natural pool with diameter ~ 110 cm, depth ~ 10.5 cm. A vertical pipe was connected to the outflow, with a bucket at the top. Lots of sinter on the pipe and bucket. Green mats on the edge of pool, Yellow mats floating on the pool. Sample types include mats, sediment, sinter and water.
DB	Danba	6.56	64.6	4000	30°36′39″ N 101°41′9″ E	A small pool with outflow from the rock wall. Green mats on the rock. Clear sulfur water. Sample type includes mats and water.
MN	Maoniugu	6.8	95	4000	30°36′9″ N 101°43′46 E″	A shallow pond with small outlet, irregular shaped with length ~ 65 cm, width ~ 45 cm, and depth ~ 5 cm. Clear water. Reddish and green mats on the edge of pond. Sample type includes sediment and water.
LL-1	Lotus lake	8.32	95	4200	29°25′4″ N 101°18′1″ E	Small natural source pool with slow outflow. Roughly shaped with length ~ 65 cm, width ~ 55 cm, and depth ~ 35 cm. Clear water and fine stone bottom. Sample type includes sediment and water.
LL-2	Lotus lake	8.41	75.2	4200	Near LL-1	Shallow small pool with spouting outlet. Length ~ 65 cm, width ~ 55 cm, and depth ~ 15 cm. Lots of reddish brown mats on the edge of spring. Many black fine stone at bottom. Sample type includes mats, sediment, and water.
LL-3	Lotus lake	8.84	41.1	4200	Close to LL-1	Shallow water flow channel (~ 55 cm width, ~ 5 cm depth) downstream of LL-1. Clear water. Many blue-green biofilm on rocks. Sample type includes biofilm and water.
LL-4	Lotus lake	8.61	42.7	4200	Close to LL-2	Shallow water flow channel (~ 85 cm width, ~ 5 cm depth) downstream of LL-2. Many green filaments. Clear water and rocky bottom. Sample type includes sediment, water and filaments.
LL-5	Lotus lake	7.95	67.2	4200	Near LL-1	Spring pool with jet outflow. Length ~ 4 m, width ~ 3 m, and depth ~ 800 cm. Black sands and fine stone at the bottom. Some yellowish-white filament on the bank of pool. Brown mats at spring outlet. Sample type includes water, sediment, filament and mats.
LL-6	Lotus lake	8.21	68.5	4200	Near LL-1	The spring outflow from cracks in stone. Irregularly shaped with length ~ 80 cm, width ~ 45 cm, and depth ~ 35 cm. Clear water. Silvery and yellow rocks at bottom. Blue-green biofilms on the edge of spring. Sample type includes biofilm, sediment and water.
LL-7	Lotus lake	8.18	68.1	4200	Near LL-1	A sulfate pool with slow outflow. Roughly rounded with diameter ~ 4 m, depth ~ 4 cm. Yellowish-white filaments and brown mats in center of pool. Black-green filament on bank of spring. Sample type includes filament, mats and water.

### DNA extraction

Genomic DNA was isolated from various sources such as microbial mats, sinter, sediments and spring water as presented in Table 1. All samples were processed within 48 h from collection and maintained at <8 °C during this period in a mobile battery-operated cooling unit before processing. For each of these samples three methods of DNA extraction were used to minimise bias of DNA isolation protocols: a modified Xantogenate method essentially as described by Avijeet Singh [29], and commercial kits: FastDNA™ SPIN Kit for Soil (MP Biomedicals), and Bacterial Genomic DNA Extraction Kit (Generay), both according to manufacturers’ instructions. The DNA quality and quantity were assessed using agarose gel electrophoresis and Nanophotometer (Impeln), respectively. The DNA isolated from different material and using different methods representative of each location were mixed in equimolar ratios. DNA concentration of each of these samples was adjusted to 100 ng μL^− 1^ before PCR amplification.

### Amplification and sequencing of targeted 16S genes

The V4-V5 region of 16S rRNA gene was amplified with universal primer pair 515F (5′-GTGCCAGCMGCCGCGG-3′) and 907R (5′-CCGTCAATTCMTTTRAGT-3′) [23] combined with Illumina adapter sequence, a 2-bp index and 8-bp barcodes specific to different samples [30]. DNA amplifications were performed using a C1000 Touch ™ thermal cycler (Bio-Rad Laboratories, USA) with 50 μL reaction mixtures containing 2.5 μL DNA (~ 10 ng/μL), 1.25 μL each primer (10 μM), 25 μL High-Fidelity PCR Master mix (GENERAY, China) and 20 μL sterile deionized water. PCR cycling conditions consisted of denaturation at 94 °C for 3 min, 30 cycles of 94 °C for 30 s, 52 °C for 30 s, and 72 °C for 40 s, and final extension at 72 °C for 10 min. PCR reactions were carried out in triplicate for each sample and amplicons of each sample were combined. Positive PCR products were separated by 1% agarose gel electrophoresis and purified using a GENERAY purification Kit (GENERAY, China). Then, PCR products were double-checked by 1% agarose gel electrophoresis and quantified using Nanodrop 1000 (Nanodrop Technologies, USA). The barcode-tagged PCR products of each sample were pooled and purified using the QIAquick PCR Purification Kit (Qiagen, Hilden, Germany). Sequencing library of 16S rRNA was constructed following the MiSeqTM Reagent Kit Preparation Guide (Illumina, USA) and the protocol described previously [28]. Finally, the amplicon library was sequenced on an Illumina MiSeq PE250 platform [23–25]. The raw sequence reads generated have been deposited into Sequence Read Archive at NCBI with accession numbers SRR5104369-5104384.

### Preprocessing, clustering and taxonomic assignment of sequence data

Raw sequences with perfect matches to barcodes were first assigned to different sample libraries using a custom Perl script. Quality control was preprocessed for each sample library to filter low quality reads and reads with N by QIIME v1.8.0 [31]. The remaining reads were considered as clean reads. Forward and reverse reads with at least 25 bp overlap and lower than 5% mismatches were assembled into tags using Fast Length Adjustment of Short reads (FLASH) [32]. The tags were subjected to chimeras screening using UCHIME [33]. Operational taxonomic units (OTUs) were clustered at a threshold of 97% similarity through UPARSE [34]. The representative sequences of each OTU were annotated with taxonomic assignment using Ribosomal Database Project (RDP) classifier as described by Zhang et al. [23].

### Diversity analysis

Alpha diversity analysis was conducted to calculate Chao1, observed species, Shannon and Simpson indices using Mothur v1.31.2 (https://mothur.org/wiki/Calculators) as described by Zhang et al. [23]. Rarefaction curves were generated based on the four indices. Beta diversity was analyzed by QIIME. Hierarchical clustering was performed using unweighted pair group method with arithmetic means (UPGMA) based on the Bray-Curtis dissimilarity matrix generated from the OTU table. Principal coordinates analysis (PCoA) was conducted to complement the output of the cluster analyses. A nonmetric multidimensional scaling (NMDS) was depicted to visualize the Bray-Curtis distances among samples using Vegan in R package (https://www.r-project.org/). Physicochemical variables were fitted onto the ordination space (Bray-Curtis NMDS) to assess correlations between environmental variables and bacterial community structure using the “envfit” function of Vegan package, and statistical significance was evaluated by 1000 permutations. Nonparametric multi-response permutation procedure (MRPP) was used to test for significant differences in bacterial communities between samples.

## Results

### Overview of sampling sites and meta-16S sequencing data

**Fig. 1:**
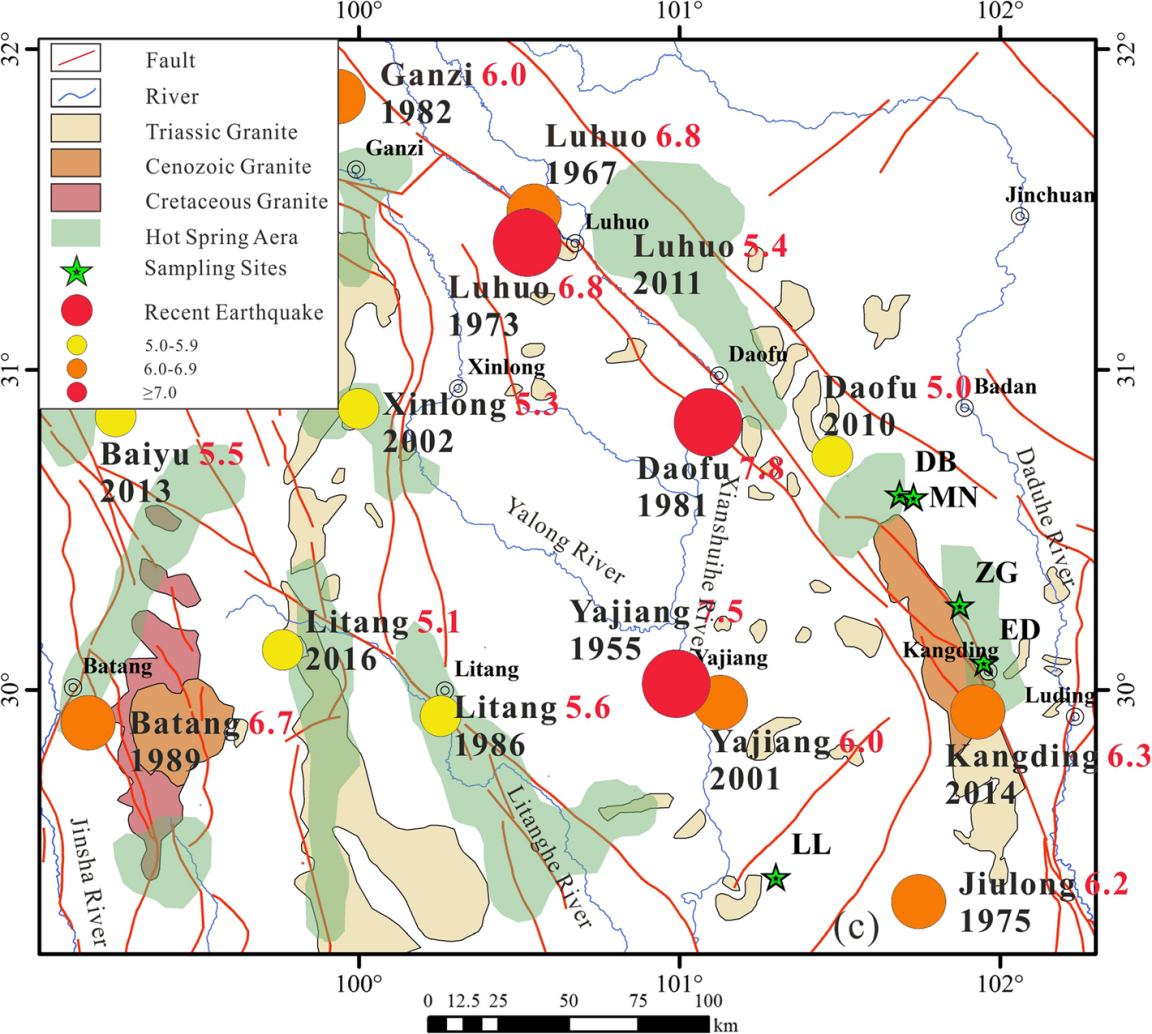
Geological settings of Western Sichuan. Sampling sites are marked with green stars, faults with red lines. Earthquakes with magnitude 7 or more are marked as red spots, magnitude 6.0–6.9 with orange, magnitude 5.0–5.9 with yellow. Earthquakes with magnitude <5.0 are not shown. For additional information about the earthquakes please consult Additional file 1: Table S1. Map has been drawn with combination of Arcgis and Corel Draw using data of Reference [11] for lithology

Fourteen springs in Ganzi were sampled for bacterial investigation. The 14 springs varied in temperature (40.8–95 °C), pH (6.32–8.84) and altitude (2600–4200 m) (Table 1). All sites except Lotus lake were situated on or in the close vicinity of Xianshuihe fault (Fig. 1, Additional file 1: Table S1) that experienced numerous earthquakes in last 300 years resulting in emergence, disappearance and radical changes of numerous hot springs in the area [11]. Lotus lake was situated in the proximity of smaller fault in a tectonically active area that experienced strong earthquakes in 1955 and 2001 (Fig. 1, Additional file 1: Table S1). Based on previous research geology of the area can be divided into two regions: north, northwest of Kangding (Kangding type geothermal belt [11], and south, southwest of Kangding (Batang-type geothermal belt [11]. The division line approximately follows the Xianshuihe fault. Sampling sites ED, DB, ZG, MN are likely to be situated in Triassic carbonate rocks, and more specifically in crystalline limestone [11] containing silicate [35]. The remaining site, LL, southwest of Kangding, is situated in the area rich in Yanshanian granite and limestone [11].

### Overview of meta-16S sequencing data

High-throughput sequencing on the Illumina MiSeq platform was performed on samples isolated from the sites described in Table 1, yielding a total of 899,830 clean reads after the quality filtration. Sequence read merging process and chimeras screening finally generated 281,979 tags, with an average length of 377 bp (Additional file 2: Table S2). Using a 97% sequence similarity cut-off, 1904 OTUs were identified in the 14 samples (Additional file 2: Table S2), on average 136 OTUs for each sample. The rarefaction curves almost reached a plateau as shown in Additional file 3: Figure S1, indicating that greater sequencing depth will not likely identify new phylotypes.

### Alpha diversity of bacterial communities

Alpha diversity analysis was performed to elucidate the species richness and diversity using four indices. As shown in Table 2, a variety of taxa were evident among the spring samples, with 133–796 observed OTUs, 202–922 predicted OTUs (based on Chao1) and coverage values ranging from 55.60–87.11%. Spring LL-4 exhibited the highest species richness, while spring LL-2 showed the highest species diversity. There’s a decline in species richness in terms of the spring temperature exceeding 65 °C. However, an exception was also noticed that spring LL-2 surprisingly had relatively high species richness at temperature as high as 75 °C. The species diversity among spring samples was not observed in their correlation with temperature pH and spring altitude.

**Table 2:** Species richness and diversity of hot springs based on alpha diversity analysis

Sample names	Observed species	Coverage value (%)	Chao1	Shannon	Simpson
ED	424	83.39	508.443	3.794	0.054
ZG-1	463	74.30	623.111	4.167	0.033
ZG-2	583	87.11	669.241	4.020	0.058
ZG-3	423	74.26	569.654	3.689	0.077
ZG-4	359	77.36	464.060	3.576	0.088
DB	413	76.85	537.385	4.367	0.024
MN	278	55.60	500.000	2.954	0.096
LL-1	206	68.31	301.577	2.219	0.335
LL-2	649	77.73	834.975	4.785	0.033
LL-3	558	80.97	689.154	2.857	0.258
LL-4	796	86.26	922.806	4.587	0.073
LL-5	271	70.60	383.857	3.309	0.097
LL-6	182	58.07	313.400	2.453	0.147
LL-7	133	65.84	202.000	2.105	0.292

Coverage value is the ratio of observed species to Chao1

### Bacterial community compositions

**Fig. 2:**
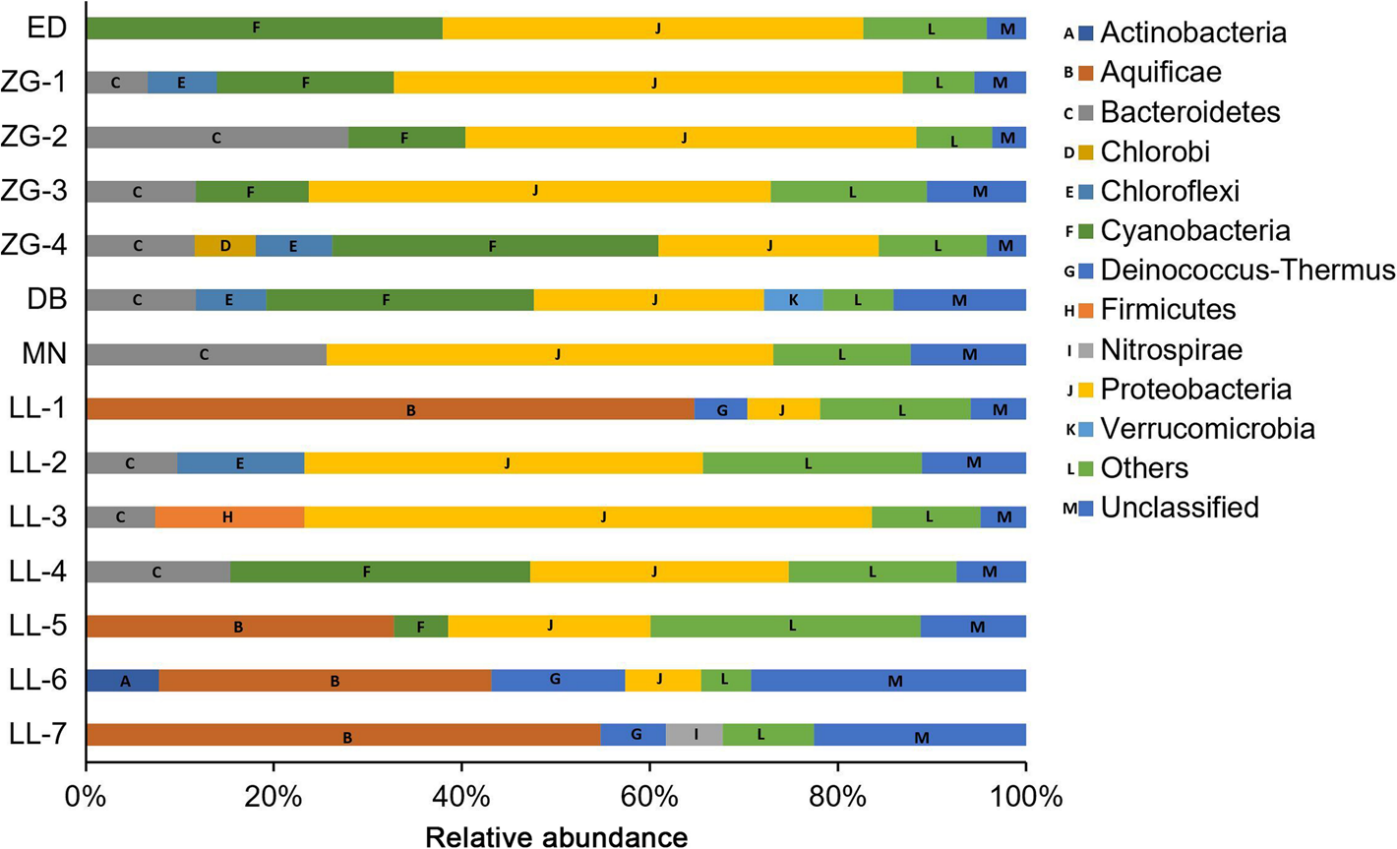
Bacterial community composition of spring samples at phylum level. Only the bacterial groups with relative abundances higher than 5.6% are displayed. The groups with relative abundances lower than 5.6% are included as “Others”

Bacterial community compositions were evidently distinct among the 14 hot springs (Fig. 2 and Additional file 4: Table S3). Among the springs, 7 out of 14 (spring ED, ZG-1, ZG-2, ZG-3, MN, LL-2 and LL-3) were dominated by *Proteobacteria* (42.39 to 60.20%), and the remaining in the bacterial communities were primarily composed of *Bacteroidetes* (11.71 to 27.89%), *Chloroflexi* (13.51%), *Cyanobacteria* (18.81 to 37.96%), and *Firmicutes* (15.89%). Spring ZG-4, DB and LL-4 mainly comprised *Cyanobacteria* (28.41 to 34.67%), *Proteobacteria* (23.42 to 27.50%) and *Bacteroidetes* (11.57 to 15.35%), while the spring LL-1, LL-5, LL-6 and LL-7 were predominated by *Aquificae* (32.76 to 64.69%), followed by *Deinococcus*-*Thermus* (6.94 to 14.24%) and *Proteobacteria* (7.76 to 21.54%). Unclassified phyla were detected in all the springs, ranging from 4.19 to 29.29%, no correlation between abundance of unclassified phyla and temperature and/or site were observed. Arguably the most interesting spring in this dataset is LL-2 (75.2 °C, pH 8.41), characterized by high species richness despite high temperature of 75.2 °C and relatively high pH. Bacterial community of this spring is composed largely of *Proteobacteria* (42.39%), *Chloroflexi* (13.51%) and *Bacteroidetes* (9.72%) at phylum level. At genus level, only six genera were present in abundances higher than 1% (Additional file 5: Table S4): *Ignavibacterium* (3.74%), *Meiothermus* (2.69%), *Tepidimonas* (1.95%), *Novosphingobium* (1.06%), *Sphingomonas* (1.06%), *Fervidobacterium* (1.02%). All these genera are typical inhabitants of hot springs. Majority of the sequences however (73.1%), could not be ascribed to any known genus what indicates a possibility of novel genera or phylotypes.

**Fig. 3:**
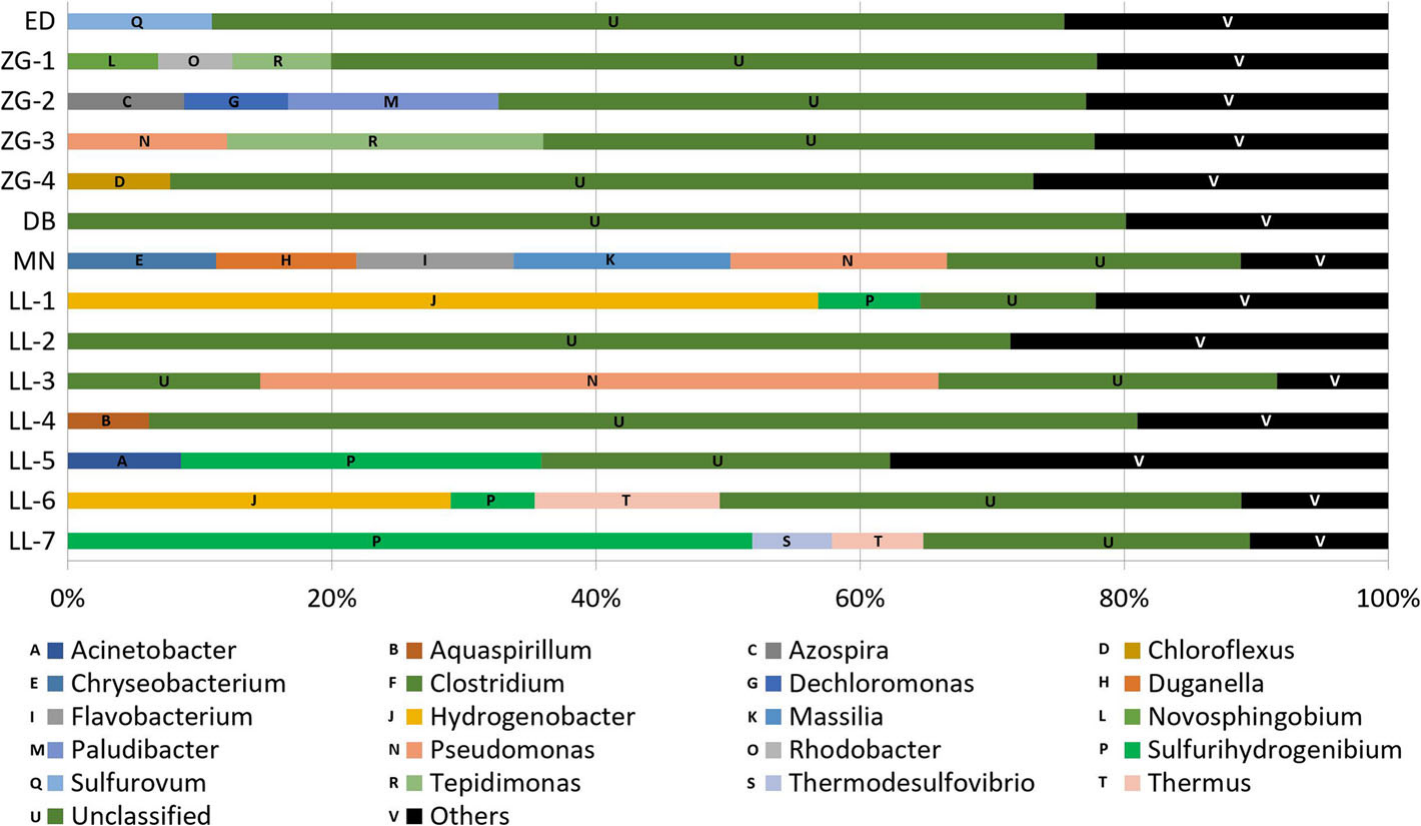
Bacterial community composition of spring samples at genus level. Only the bacterial groups with relative abundances higher than 5.6% are displayed. The groups with relative abundances lower than 5.6% are included as “Others”

As showed in Fig. 3 and Additional file 5: Table S4, at the genus level, spring LL-1 and LL-3 was largely harbored by *Hydrogenobacter* (56.82%) and *Pseudomonas* (51.38%), respectively, whereas spring LL-5 and LL-7 was abundant in *Sulfurihydrogenibium* (27.25 and 51.85%). However, the rest springs were mainly composed of sequences that did not belong to any known genus, ranging from 22.25 to 80.15%. Most of the unknown-genus sequences were from class *Alphaproteobacteria*, *Betaproteobacteria* and *Cyanobacteria*.

### Clustering and correlation analysis of bacterial community

**Fig. 4:**
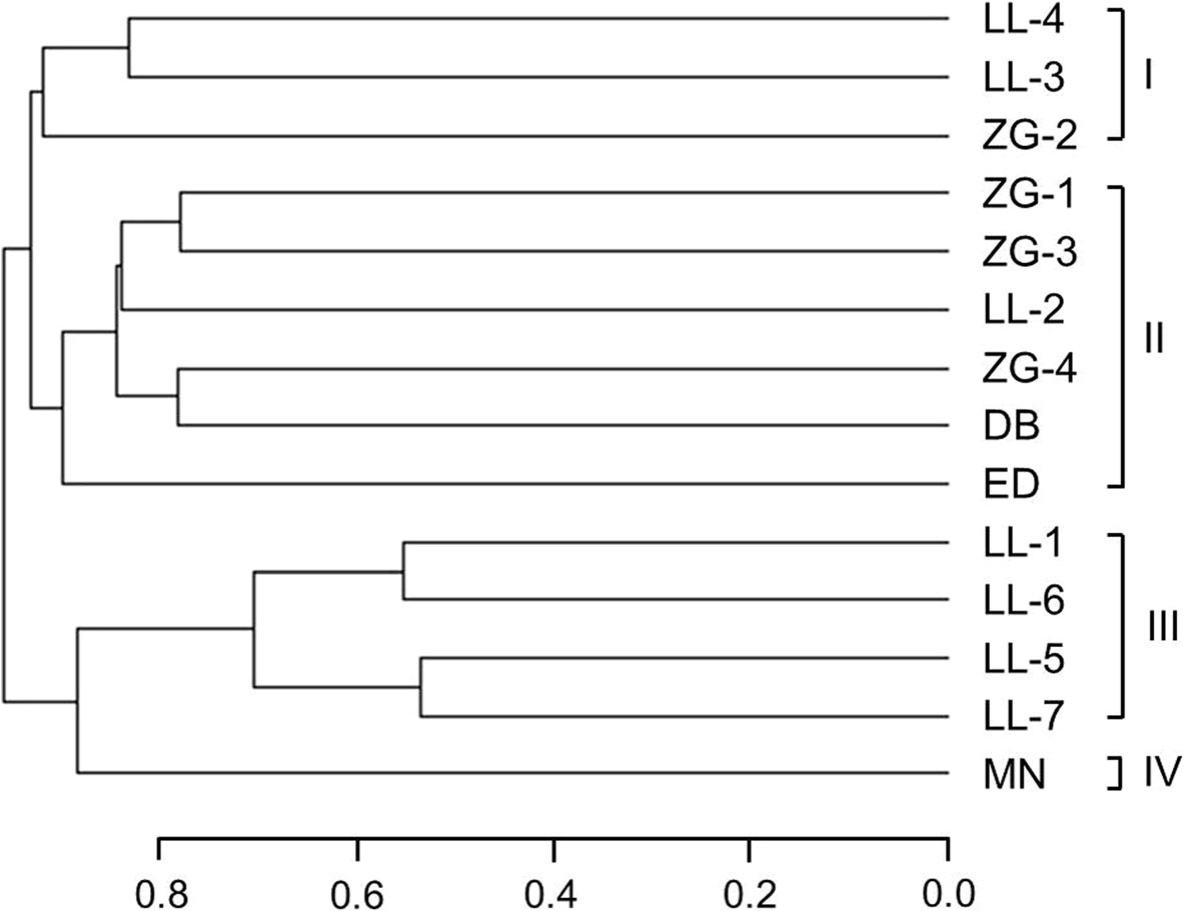
Cluster analysis of bacterial community in spring samples based on Bray-Curtis dissimilarity at the 97% OTU level

Based on Bray-Curtis dissimilarity at the 97% OTU level, an UPGMA cluster tree of the bacterial community of spring samples was constructed (Fig. 4). The clustering result indicated that 14 spring samples were categorized into four bacterial community patterns. Pattern I included three spring samples with low temperature (≤ 55 °C) and neutral-high pH (6.6–8.8), while pattern II showed a mixed sample types, the temperature and pH of which ranged from 41 to 85 °C and from 6.3 to 8.5, respectively. Spring samples belonging to pattern III all exhibited high pH (≥ 8.0) and high temperature (>65°C). Only one spring sample was affiliated to pattern IV, which showed a moderate temperature and neutral pH.

**Fig. 5:**
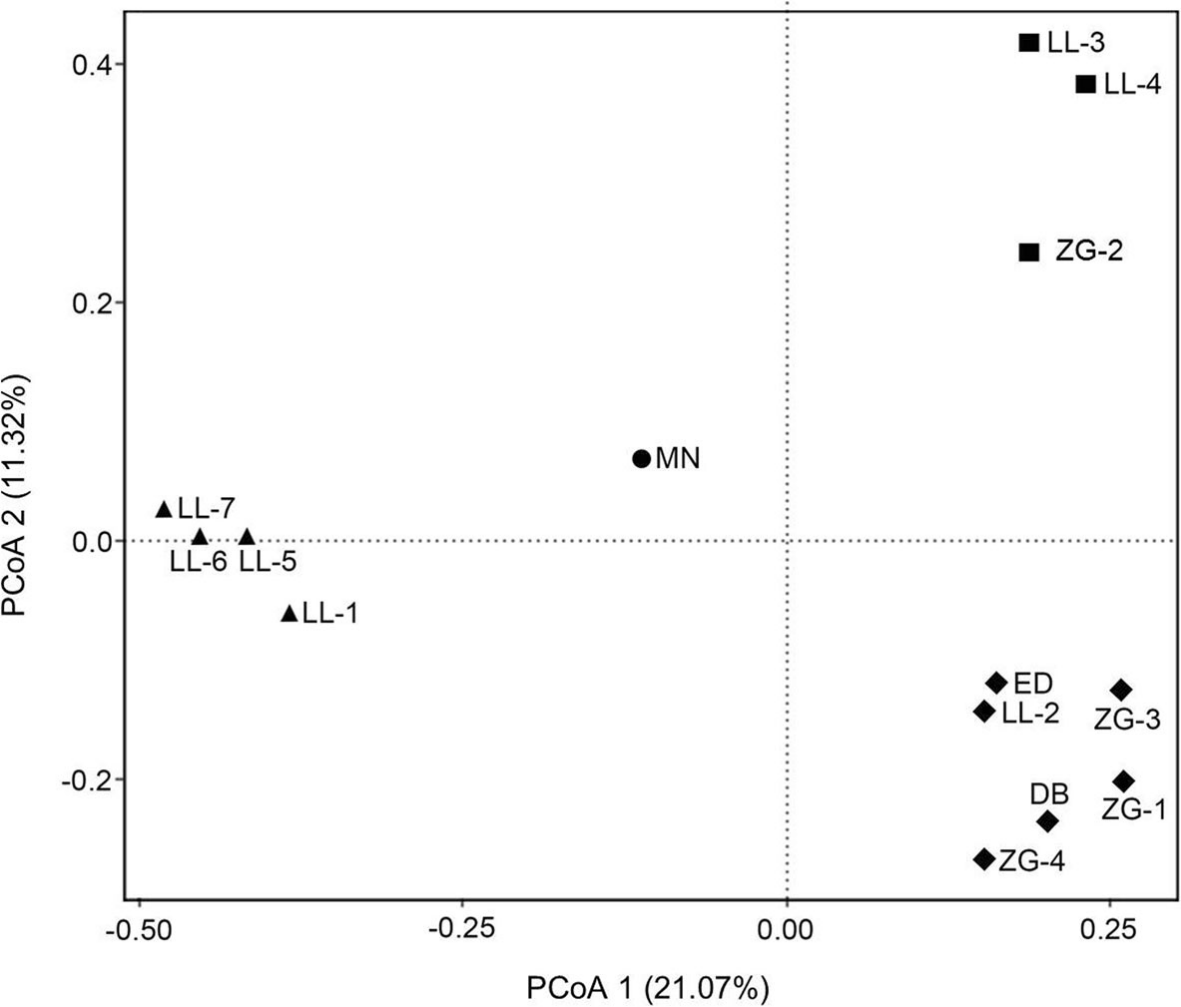
Principal coordinate analysis (PCoA) of bacterial community patterns in spring samples. Spring samples belonging to Pattern I to IV are represented by: closed squares (I), closed rhombi (II), closed triangles (III), and closed circles (IV), respectively

PCoA analysis was also conducted as a complement of cluster analysis to visualize the patterns of bacterial community. The PCoA analysis is consistent with the cluster analysis, confirming the four bacterial community patterns in the spring samples (Fig. 5). The two dimensions of PCoA accounted for 21.07 and 11.32% of the diversity variations, respectively.

**Fig. 6:**
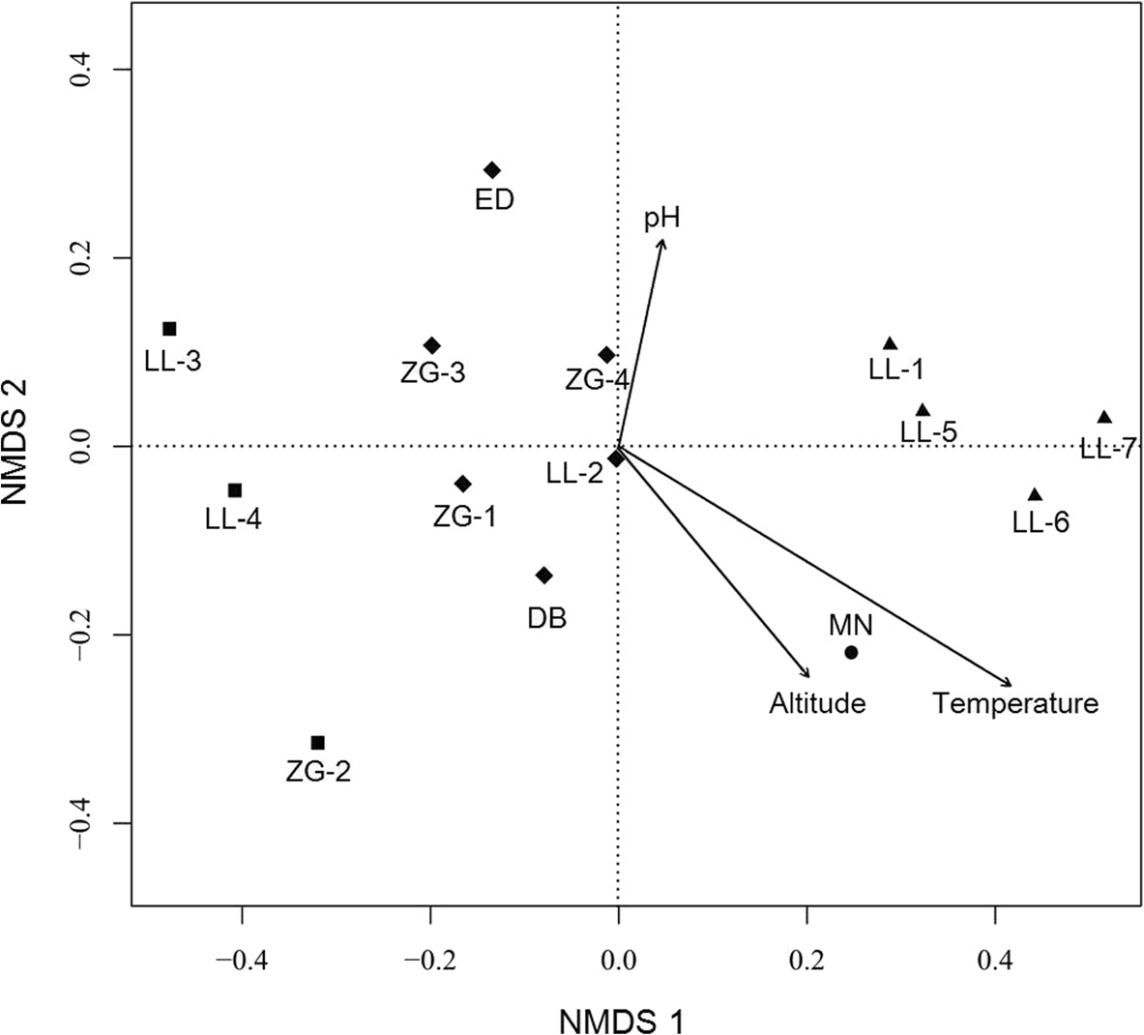
Non-metric multidimensional scaling (NMDS) ordination for the community structure of all the spring samples

The correlation between bacterial community structure and three geochemical parameters were investigated by Envfit function implemented in Vegan. The results indicated that temperature with *R*^2^ value of 0.47 was significantly correlated (*P* < 0.05) with bacterial community structure (Fig. 6), suggesting that temperature, as a driving force at least, was responsible for shaping bacterial community structure in hot springs. However, no significant correlation was observed between pH (*R*^2^ = 0.10) or altitude (*R*^2^ = 0.20) and bacterial community structure.

## Discussion

In the present study, the bacterial communities of 14 hot springs distributed in Western Sichuan, China, were investigated for the first time through molecular examination. Different analyses were conducted to elucidate the diversity of each hot spring. All clustering analysis supported to categorize the 14 spring samples into four bacterial community groups. Correlation between geochemical parameters and bacterial community structure was also studied.

The Alpha diversity analysis revealed that the values of four indices greatly varied among spring samples (Table 2). This finding could be explained mostly by the distinct temperature between the springs (Table 1). It is known that temperature is an important determinant of bacterial diversity [5]. Normally, microbial richness/diversity showed a positive or negative liner correlation with temperature [2, 36]. Such linear correlation, however, was not detected in this study, what has been also observed in many studies before [36–38]. These results indicate that although very important, temperature is not an exclusive determinant of microbial diversity in these hot springs. A remarkable decline in species richness was observed when the spring temperature exceeded 65 °C. Simultaneously, with an increase of temperature cyanobacteria become less abundant, green microbial mats were absent and typical hyperthermophilic organisms such as *Aquificae* and certain thermophilic members of *Proteobacteria* became more abundant. The disappearance of cyanobacteria, especially above 75 °C, is in agreement with previous studies that indicate that oxygenic photosynthesis is possible up to approximately 73–75 °C [39]. Above these temperatures, even the most thermophilic cyanobacteria described to date i.e. *Synechococcus* sp. OH28 isolated from Hunter’s Hot Springs in Oregon, USA [39] cannot actively grow. Although one of the springs indicated in this study ZG-4 (85.0 °C, pH 8.5) shows significant contribution of cyanobacterial population to the overall community structure, it is likely the result of sequencing approach used in the study. The 16S PCR products originating from DNA samples isolated from sediments and microbial mats collected from different parts of the spring were mixed together before sequencing. The cyanobacterial sequences are likely to originate from cooler parts of the spring and it is those sequences that contributed cyanobacterial part of the diversity. This has been confirmed by our parallel culture-dependent study that showed that although strains isolated from ZG-4 (equivalent to B4 strain series in that study) were among the most thermophilic ones and capable of growth at 60 °C they were unable to actively grow at temperatures higher than 65 °C [17].

An exception to the overall trend of decreasing microbial diversity as a response to increase of spring temperature is spring LL-2 (75.2 °C, pH 8.41) containing numerous reddish microbial mats. Detailed comparative analysis of the community composition of this spring is problematic due to very high percentage (73.16%) of unassigned at genus level sequences. At phylum level the spring is mainly composed of *Proteobacteria* (42.39%), *Chloroflexi* (13.51%), and *Bacteroidetes* (9.72%). Comparison of LL-2 spring with other hot springs of similar temperature and pH reveals both similarities and differences. The community structure of LL-2 is most similar to the recently described Mihai-Bravu (MB) spring in Romania (65 °C, pH 7.91) [40]. The MB spring sampled at 65 °C was dominated by *Proteobacteria*, *Aquificae* and *Chloroflexi* with minor contribution of *Bacteroidetes* making the community similar to that of LL-2. These results suggest that these communities may be sustained by anoxygenic photosynthesis, oxidation of hydrogen or combination of both factors [40].

One of the springs that has similar parameters to LL-2 is bicarbonate-chloride-fluoride low mineral-type Lobios hot spring (76 °C, pH = 8.2) located in Ourense (Spain) [41]. The spring is predominantly composed of bacteria: *Deinococcus-Thermus*, *Proteobacteria*, *Firmicutes*, *Acidobacteria*, *Aquificae*, and *Chloroflexi*. This alkaline spring is therefore inhabited by thermophilic, neutrophilic, and alkaliphilic organisms which belong to both aerobes and anaerobes with the largest abundance of *Thermus* species. It therefore shows different community structure to LL-2. Another spring similar in pH and temperature described in the literature is a sulfate-bicarbonate spring in Rupi Basin, Bulgaria [42]. The temperature and pH of the spring were 79 °C and 8.6, respectively. This spring was dominated by *Proteobacteria* (28.8%), *Hydrogenobacter*/*Aquifex* group (26.3%) and *Deinococcus-Thermus* (19.5%). Community structure of the Bulgarian spring suggested that metabolic activities are likely to be distinct of those of LL-2. Rupi Basin spring is likely to be shaped through co-existence of hydrogen-oxidizing *Hydrogenophilus* and *Hydrogenobacter/Aquifex* with hydrogen producing *Thermus* and *Thermotoga*, indicating hydrogen metabolism as an important component of that ecosystem. A similar type of microbial community was present in sulfuric hot spring Scary Spring (pH 7.8, 75 °C), Calcite Springs, YNP. Scary Spring has a very simple community structure composed predominantly of *Thermus aquaticus* (45%) and *Sulfurihydrogenibium* sp. (36%) [43], largely distinct than that of LL-2. One of three alkaline springs from Heart Lake Geyser Basin, Western Subgroup in YNP, that have similar basic parameters (pH 8.5, 75 °C) to LL-2 have been also assessed for their community structure [44] and showed to be different from that of Lotus lake spring. The microbial community of the spring is largely dominated by *Thermus*, analogously to the previous springs described above. In China, an alkaline spring Shuirebaozha in Tengchong region, Yunnan has most similar pH and temperature (pH 8.28, 78.2 °C) to LL-2 [6]. The bacterial community in the spring is dominated by *Aquificae* with secondary contributions from *Chloroflexi* and *Thermotogae* indicating very distinct composition from that of LL-2. All of the springs described in this section have lower species richness and diversity indices than that of LL-2.

To summarize, LL-2 has largely atypical community structure, and unusual biodiversity when compared to other springs with similar temperature and pH worldwide. The reasons of that can be many-fold and there is not enough evidence in the current dataset to make final conclusions. Some of the possible explanations are presented below. Different mineralogy to other springs in the area; it has been shown that hot springs even in close proximity can significantly differ in mineralogy, pH and temperature [6]. Water flows and temperature change patterns; unlike well-known springs of YNP and Tengchong, and even Danba (DB) or Erdaoqiao (ED) presented in this study Lotus Lake springs are remote and largely unknown to scientific community and only visited by autochthonic Tibetan peoples. The water flow patterns and temperature changes are largely unknown so one cannot accurately describe the history of the spring. We take into consideration the fact that the spring may experience a degree of temperature fluctuations that result in formation of more diverse community than typically found for springs of this temperature. Both temperature and flow pattern fluctuations have been reported for hot springs previously and are considered as important contributors to biodiversity and community structure [40, 45]. Another possibility is the carryover of non-endemic material e.g. soil from outside the pool to the pool itself. Since genomic DNA was used as a template for 16S rRNA amplification in this study, there exists a possibility that even if the biological material cannot survive harsh conditions of the spring the genomic DNA can remain stable and yield over-representation of certain genera non-endemic to the spring. A study performed using both genomic DNA and RNA (cDNA) as a template for 16S amplification combined with soil sampling around the pool could be used to verify this possibility as proposed before [40]. Whilst all these hypotheses provide possible explanations of unusually high diversity of the spring they explain very little as to why almost three quarters (73.16%) of sequences originating from this spring cannot be classified at genus level. We believe that more detailed follow-up study of Lotus Lake springs will be required to solve at least some of these issues.

To summarize temperature effect on Ganzi hot springs, there is a non-monotonic relationship between microbial diversity and temperature of the springs assayed. Similar findings were reported in previous studies [8, 37, 46], and it is likely that albeit very important, the temperature is not a unique determinant of microbial diversity in the hot springs of Ganzi.

pH of the springs analyzed in this study is in relatively narrow pH range: from slightly acidic (ED, pH 6.32) to moderately alkaline (LL-3, pH 8.84). Extreme pH ranges are well known to significantly impact the biodiversity to the point where the biological niche is occupied only by several most adapted species. For example, acid mine drainage site in Iron Mountain, California, USA (pH 0.83, 42 °C) is inhabited by a very simple community dominated by an extremophilic *Leptospirillum* and *Ferroplasma* [47]. One of the best studied acidic hot springs are those of Diretiyanqu in Rehai, Tengchong, Yunnan, China [6]. Diversity of these springs is lower than those of more neutral pH and temperature dependent. For example, Diretiyanqu-1 spring (pH 2.58, 85.1 °C) is almost exclusively inhabited by archaeal strain belonging to *Crenarchaeota*, whilst colder Diretiyanqu-2 (pH 2.57, 64.5 °C) and Diretiyanqu-3 (pH 2.58, 55.1 °C) springs contain significant proportions of *Aquificae* and *Proteobacteria*. At alkaline pH range the effect is similar i.e. low biodiversity is observed only in large extremes, of both pH and temperature whilst moderately alkaline (pH 8.5–10) sites have comparable biodiversity to those of more neutral pH. For example, a study of hot springs in Jiemeiquan (pH 9.25, 93.6 °C) and Gumingquan (pH 9.36, 82.5 °C) in Rehai, Tengchong, Yunnan, China [6] revealed dominance of *Aquificae* over any other phyla. Typically lower temperature alkaline hot springs below 73 °C are typically dominated by cyanobacteria [39]. The decrease of microbial diversity is minor when compared with sites of neutral pH and similar temperature. Though no obvious correlation was observed between pH and diversity indices, previous studies implied that pH played important role in microbial diversity [5]. The effects are both direct and indirect since pH has an effect on mineralogy, acidic springs result in leaching metals like iron from the rocks [45] and alkaline springs affect the availability of bicarbonate for photosynthetic cyanobacteria [48]. The effect of pH on biodiversity of the hot springs is therefore more subtle than that of temperature. Although there is no statistical significance between diversity indices and pH, we have observed that these hot springs are hosts to some alkaliphilic cyanobacteria which can actively grow in pH exceeding 9.50 [17], which corresponds to the fact that geologically these hot spring are likely to be limestone-hosted [11]. Taken together, these results indicated that differences in richness/diversity could be ascribed to a combined impact of various environmental conditions, such as temperature, pH and mineralogy [6, 36].

The bacterial communities in hot springs of Ganzi Prefecture were abundant in phyla *Aquificae*, *Cyanobacteria* and *Proteobacteria* (Fig. 2 and Additional file 4: Table S3). Although all the hot springs in this study were located at high altitude (2600–4200 m), the composition of bacterial community were common to that of low-altitude springs and does not appear to be unique to high-altitude springs. This result is consistent with previous finding in Tibetan springs (around 4500 m) [8, 37]. Differences in bacterial composition were also found between Ganzi hot springs and other geothermal springs. For instance, *Thermodesulfobacteria* were found to be abundant in springs of Tengchong, YNP and Great Basin [6, 49, 50]. However, low relative abundance of *Thermodesulfobacteria* was observed in Ganzi springs, with the exception of LL-7 where strong sulfur smell was observed (Table 1, Additional file 4: Table S3). This is probably because of the unfavorable geochemical conditions in Ganzi springs for *Thermodesulfobacteria* that showed the common presence in neutral pH (6.1–7.3) and high temperature (77–90 °C).

Within phyla *Aquificae*, *Hydrogenobacter* and *Sulfurihydrogenibium* were predominant genus. The two genera co-existed in several springs (Additional file 5: Table S4), which is consistent with observations in Tibetan and YNP hot springs under similar pH and temperature (i.e., near neutral to slightly alkaline pH and a temperature range of 60–80 °C) [8, 51]. Interestingly, the co-existence occurred in both sulfate pool (LL-7) and low-sulfate pool (LL-5) (Table 1). This result confirmed the previous finding that sulfide concentration is not a limiting factor for the co-existence of the two genera [8]. Unexpectedly, the co-existence was also noticed in spring LL-1 (Additional file 5: Table S4) which has a high temperature of 95 °C and high pH of 8.3. The possible reason may be that microorganisms in the spring may have distinct physiological properties. Additionally, the prominent *Aquificae* in springs indicated that chemolithotrophy by oxidation of H_2_, reduced sulfur compounds (sulfur or sulfide), or one-carbon compounds (formate or formaldehyde) are likely important metabolic processes in these springs [6].

Cyanobacterial sequences were abundant in spring ED, ZG-4 and DB (Fig. 2) and were dominated (72.37–99.67%) by members that could not be assigned below the class level (Additional file 5: Table S4). This result indicated the presence of unique members within the cyanobacterial taxonomy and yet unknown ecological role of these prokaryotic members. This is confirmed by our recent culture-dependent study of cyanobacteria from these springs where four out of five potential novel phylotypes of Leptolyngbyaceae were identified, the fifth one being LL [17]. Spring LL-4 was also abundant in cyanobacterial sequences, majority of which (81.13%) were affiliated with family GpXIII (Additional file 5: Table S4). The other cyanobacterial sequences detected in this study were related to groups (GpI, GpIIa, GpIV, GpV, GpVI, GpIX and GpXI) and families (*Bacillariophyta*, *Chlorophyta* and *Streptophyta*) (Additional file 5: Table S4). The classification scheme used in this study followed the RDP classifier [52], which is based on the classification described in Bergey’s Manual of Systematic Bacteriology. Unfortunately, this scheme failed to describe many Cyanobacteria beyond the family level as indicated above. Similar results were achieved in previous studies [53, 54]. Furthermore, cyanobacterial taxonomy was historically based on morphologies according to the Botanical Code, whereas their 16S rRNA sequence-based taxonomy is hampered by incorrect naming [55]. Thus, a high resolution of cyanobacteria taxonomy will require dedicated work in future on accurate systematic nomenclature and corresponding sequence database.

As mentioned above, seven springs were dominated by *Proteobacteria* (Fig. 2). This bacterial composition is commonly observed in some hot springs [56–58], but also somehow different than YNP and Tengchong hot springs [6, 59]. Class *Alphaproteobacteria*, *Betaproteobacteria*, *Deltaproteobacteria*, *Epsilonproteobacteria* and *Gammaproteobacteria* were detected in all the seven samples but with variable abundance. Genus *Tepidimonas* of *Betaproteobacteria* was predominated within *Proteobacteria* in spring ZG-3. Genus *Dechloromonas* of *Betaproteobacteria* was abundant within *Proteobacteria* in spring ZG-2, which represents a unique genus with a broad range of novel metabolic capabilities and bioremediative [56]. Sulfate-reducing bacteria (SRB) of the class *Deltaproteobacteria* and *Gammaproteobacteria* were also widely detected in the present study, indicating detoxification role of these bacteria as part of sulfur cycle [60].

As shown in Fig. 6, NMDS ordination showed that temperature significantly shaped bacterial community structure (*R*^2^ = 0.47, *P* < 0.05). Distinct differences were observed in bacterial composition between high temperature (65–95 °C) and low temperature (41–65 °C) groups (Fig. 2 and Additional file 4: Table S3). The majority of the sequences in high temperature group were affiliated to *Aquificae* and *Proteobacteria*, while the low temperature group was dominated by *Proteobacteria* and *Cyanobacteria*. Although statistical analysis suggested that pH did not play significant role in shaping bacterial community structure in this study (*R*^2^ = 0.10, *P* > 0.05), numerous previous studies indicated that pH is responsible for bacterial community structure. The case in the present study can be probably ascribed to a narrow and largely non-selective span of pH range (6.3–8.8). The statistical analysis also showed that altitude was not significantly correlated with bacterial community structure in hot springs (*R*^2^ = 0.20, *P* > 0.05). This result is consistent with the previous studies in Tibet and India [37, 61]. A possible cause to the case in this study is that the spring temperature is evidently higher than the environmental temperature shifts caused by altitude, leading to a dominant influence on the bacterial community structure as indicated by the correlation analysis (Fig. 6).

Abundant unassigned-genus sequences were detected in this study, especially in LL-2 hot spring. That creates potential for existence of novel genera in these sites. Our recent culture-dependent study of cyanobacteria in these hot springs revealed existence of five phylotypes that were identified as putative new species related to *Leptolyngbya* or new genera [17]. Novel genera in these springs could be shaped by either of, or combination of two selective pressures: prolonged pressure of temperature (press), and extreme environmental perturbations (pulse) caused by frequent earthquakes. It remains to be seen how many of these lineages are novel and how will the communities change over time, but hot-springs of Ganzi remain one of interesting sites to study evolution of microbial populations shaped by extreme events for the years to come.

## Conclusions

This is the first bacterial census of hot springs located in Western Sichuan, China, providing first insight into the microbial diversity in these geothermal fields. This study also highlighted the importance of temperature in shaping bacterial community structure of hot springs in Sichuan. Furthermore, abundant unassigned-genus sequences detected in this study strongly implied the presence of novel genera or genetic resources in these hot springs probably shaped by frequent tectonic activities.

## Additional files

Additional file 1: **Table S1.** Recent earthquakes in Ganzi prefecture. (DOCX 36 kb)

Additional file 2: **Table S2.** Statistical summary of sequencing by MiSeq platform. (DOCX 35 kb)

Additional file 3: **Figure S1.** Rarefaction curves. (DOCX 106 kb)

Additional file 4: **Table S3.** The relative abundances (%) at phylum level in different samples. (DOCX 42 kb)

Additional file 5: **Table S4.** The relative abundances (%) at genus level in different sample (DOCX 44 kb)

## Abbreviations

MRPP: Nonparametric multi-response permutation procedure
NCBI: National Centre for Biotechnology Information
NMDS: Non-metric multidimensional scaling
OTU: Operational taxonomic units
PCoA: Principal coordinate analysis
RDP: Ribosomal Database Project
UPGMA: Unweighted pair group method with arithmetic means
YNP: Yellowstone National Park
