# Supplementary material for: Temperature-controlled thermophilic bacterial communities in hot springs of western Sichuan, China
Source: BMC Microbiol. 2018 Oct 17;18:134. doi: 10.1186/s12866-018-1271-z (PMC6191902; doi:10.1186/s12866-018-1271-z)
Supplement: Supplementary file 1 — Table S1. Recent earthquakes in Ganzi prefecture. (DOCX 36 kb) [file 12866_2018_1271_MOESM1_ESM.docx]

**Additional file 1: Table S1. Recent earthquakes in Ganzi prefecture.**

| Earthquake time | Latitude (°) | Longitude (°) | Depth (km) | Magnitude (M) | Location |
| --- | --- | --- | --- | --- | --- |
| 14/04/1955 09:29 | 30 | 101.8 | 0 | 7.5 | Sichuan Kangding |
| 30/08/1967 12:22 | 31.6 | 100.3 | 0 | 6.8 | Sichuan Luhuo |
| 08/02/1973 00:06 | 31.6 | 100.5 | 0 | 6 | Sichuan Luhuo |
| 15/06/1974 18:19 | 31.6 | 99.9 | 13 | 5 | Sichuan Ganzi |
| 15/01/1975 19:34 | 29.4 | 101.9 | 25 | 6.2 | Sichuan Jiulong |
| 06/11/1979 03:16 | 30.6 | 99.3 | 0 | 5 | Sichuan Batang |
| 24/01/1981 05:13 | 31 | 101.1 | 12 | 6.9 | Sichuan Daofu |
| 16/06/1982 07:24 | 32 | 100 | 15 | 6 | Sichuan Ganzi |
| 07/08/1986 03:55 | 29.3 | 100.9 | 11 | 5.5 | Sichuan Litang |
| 02/06/1988 14:11 | 30.6 | 101.5 | 9 | 5 | Sichuan Daofu |
| 21/07/1989 11:09 | 30 | 99.5 | 13 | 6.7 | Sichuan Batang |
| 23/02/2001 08:09 | 29.4 | 101.2 | 15 | 6 | Sichuan Yajiang |
| 08/08/2002 19:42 | 30.9 | 100 | 0 | 5.3 | Sichuan Xinlong |
| 28/04/2010 04:22 | 30.6 | 101.5 | 8 | 5 | Sichuan Daofu |
| 10/04/2011 17:02 | 31.3 | 100.8 | 10 | 5.4 | Sichuan Luhuo |
| 18/01/2013 20:42 | 31 | 99.4 | 1 | 5.5 | Sichuan Baiyu |
| 22/11/2014 16:55 | 30.3 | 101.7 | 18 | 6.3 | Sichuan Kangding |
| 11/05/2016 17:01 | 31.3 | 100.8 | 18 | 3.9 | Sichuan Luhuo |
| 23/09/2016 01:23 | 30.1 | 99.6 | 16 | 5.1 | Sichuan Litang |
