# Supplementary material for: Temperature-controlled thermophilic bacterial communities in hot springs of western Sichuan, China
Source: BMC Microbiol. 2018 Oct 17;18:134. doi: 10.1186/s12866-018-1271-z (PMC6191902; doi:10.1186/s12866-018-1271-z)
Supplement: Supplementary file 2 — Table S2. Statistical summary of sequencing by MiSeq platform. (DOCX 35 kb) [file 12866_2018_1271_MOESM2_ESM.docx]

**Additional file 2: Table S2. Statistical summary of sequencing by MiSeq platform.**

| Sample No. | Clean reads obtained | Tags | Average length of Tags | OTU |
| --- | --- | --- | --- | --- |
| ED | 31819*2 | 21615 | 378 | 424 |
| ZG-1 | 31857*2 | 19359 | 377 | 463 |
| ZG-2 | 31662*2 | 20597 | 377 | 583 |
| ZG-3 | 31888*2 | 20343 | 377 | 423 |
| ZG-4 | 32281*2 | 21271 | 377 | 359 |
| DB | 31995*2 | 20682 | 377 | 413 |
| MN | 34978*2 | 20260 | 376 | 278 |
| LL-1 | 31249*2 | 19989 | 374 | 206 |
| LL-2 | 31429*2 | 17034 | 377 | 649 |
| LL-3 | 32129*2 | 22326 | 377 | 558 |
| LL-4 | 31802*2 | 19039 | 377 | 796 |
| LL-5 | 32294*2 | 20079 | 377 | 271 |
| LL-6 | 31891*2 | 18823 | 376 | 182 |
| LL-7 | 32641*2 | 20562 | 377 | 133 |
| Total | 899,830 | 281,979 |  |  |
