# Supplementary figures and images for: Temperature-controlled thermophilic bacterial communities in hot springs of western Sichuan, China
Source: BMC Microbiol. 2018 Oct 17;18:134. doi: 10.1186/s12866-018-1271-z (PMC6191902; doi:10.1186/s12866-018-1271-z)

**Additional file 3: Figure S1 Rarefaction curves.**

**
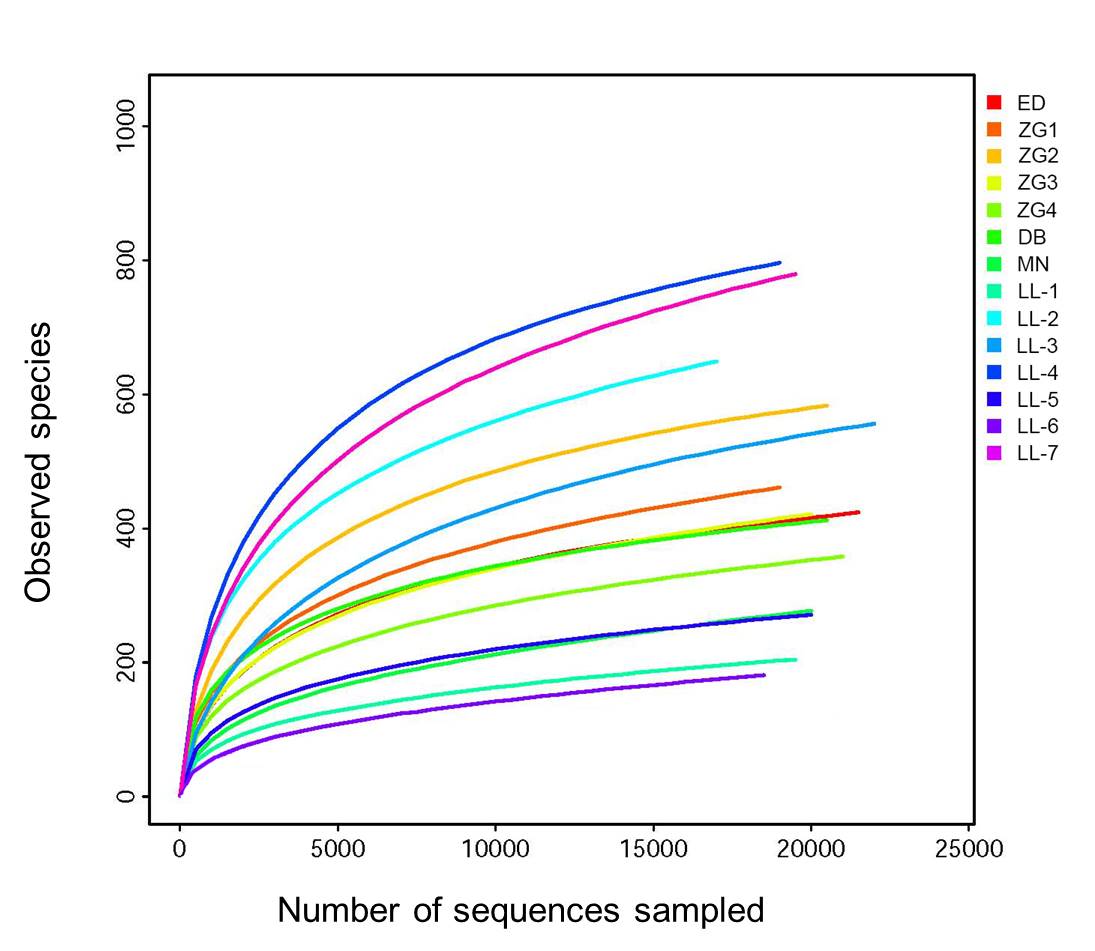
**

Supplement: Supplementary file 3 — Figure S1. Rarefaction curves. (DOCX 106 kb) [file 12866_2018_1271_MOESM3_ESM.docx]
