# Supplementary material for: Temperature-controlled thermophilic bacterial communities in hot springs of western Sichuan, China
Source: BMC Microbiol. 2018 Oct 17;18:134. doi: 10.1186/s12866-018-1271-z (PMC6191902; doi:10.1186/s12866-018-1271-z)
Supplement: Supplementary file 4 — Table S3. The relative abundances (%) at phylum level in different samples. (DOCX 42 kb) [file 12866_2018_1271_MOESM4_ESM.docx]

**Additional file 4: Table S3. The relative abundances (%) at phylum level in different samples.**

|  | **ED** | **ZG-1** | **ZG-2** | **ZG-3** | **ZG-4** | **DB** | **MN** | **LL-1** | **LL-2** | **LL-3** | **LL-4** | **LL-5** | **LL-6** | **LL-7** |
| --- | --- | --- | --- | --- | --- | --- | --- | --- | --- | --- | --- | --- | --- | --- |
| Acetothermia | 0 | 0 | 0 | 0 | 0 | 0 | 0 | 0.7 | 0.05 | 0 | 0 | 0.31 | 0.05 | 0.56 |
| Acidobacteria | 0.18 | 0.58 | 0.67 | 2.77 | 0.27 | 0.42 | 0.1 | 0.1 | 1.54 | 1.29 | 2.6 | 0.17 | 0.02 | 0.14 |
| Actinobacteria | 0.22 | 0.4 | 0.1 | 0.16 | 0.04 | 0.1 | 0.04 | 0.13 | 1.35 | 0.1 | 0.07 | 4.43 | 7.79 | 0.05 |
| Aminicenantes | 0 | 0 | 0 | 0 | 0 | 0 | 0 | 0 | 0.16 | 0 | 0 | 0.02 | 0 | 0.04 |
| Aquificae | 0.12 | 0.53 | 0.03 | 0.69 | 2.01 | 0.12 | 2.32 | 64.69 | 0.73 | 0.06 | 0.05 | 32.76 | 35.34 | 54.76 |
| Armatimonadetes | 0.06 | 0.33 | 0.08 | 0.39 | 3.12 | 0.17 | 1.03 | 3.16 | 1.04 | 0.09 | 0.14 | 1 | 1.72 | 3.82 |
| Atribacteria | 0 | 0 | 0 | 0 | 0 | 0 | 0 | 0.22 | 0.03 | 0 | 0 | 0.16 | 0.01 | 0.31 |
| Bacteroidetes | 5.27 | 6.56 | 27.89 | 11.71 | 11.57 | 11.7 | 25.64 | 0.53 | 9.72 | 7.35 | 15.35 | 3.05 | 0.18 | 0.23 |
| BRC1 | 0 | 0 | 0 | 0 | 0 | 0.02 | 0 | 0.01 | 0.25 | 0.01 | 0.01 | 0 | 0 | 0 |
| candidate division WPS | 0 | 0.03 | 0.36 | 0.13 | 0.38 | 0.01 | 0 | 0 | 0.28 | 0.09 | 0.09 | 0 | 0 | 0 |
| Candidatus Saccharibacteria | 0 | 0 | 0 | 0 | 0 | 0 | 0 | 0 | 0 | 0 | 0.1 | 0 | 0 | 0 |
| Chlamydiae | 0 | 0 | 0 | 0 | 0 | 0 | 0 | 0 | 0 | 0.01 | 0 | 0.01 | 0 | 0 |
| Chlorobi | 2.57 | 0.04 | 0 | 5.17 | 6.49 | 2.6 | 5.04 | 0.01 | 0.12 | 0 | 0.01 | 0 | 0.06 | 0.01 |
| Chloroflexi | 3.03 | 7.37 | 2.05 | 3.32 | 8.17 | 7.51 | 2.6 | 1.16 | 13.51 | 0.4 | 1.24 | 0.71 | 0.17 | 0.64 |
| Cyanobacteria | 37.96 | 18.81 | 12.51 | 12 | 34.67 | 28.41 | 0.95 | 2.62 | 4.78 | 1.94 | 31.91 | 5.77 | 0.75 | 0.03 |
| Deferribacteres | 0.13 | 0 | 0 | 0 | 0 | 0 | 0.02 | 0.02 | 0.13 | 0 | 0 | 0.01 | 0 | 0 |
| Deinococcus-Thermus | 0.12 | 0.48 | 0.01 | 0.61 | 3.29 | 0.04 | 0.23 | 5.65 | 3.38 | 0.12 | 0.19 | 4.59 | 14.24 | 6.94 |
| Dictyoglomi | 0 | 0.01 | 0 | 0 | 0 | 0 | 0.01 | 1.97 | 0.02 | 0 | 0 | 0.49 | 0.02 | 1.34 |
| Elusimicrobia | 0 | 0 | 0 | 0.06 | 0 | 0 | 0 | 0 | 0 | 0.01 | 0 | 0 | 0 | 0 |
| Fibrobacteres | 0 | 0 | 0.07 | 0 | 0 | 0 | 0 | 0 | 0 | 0 | 0 | 0 | 0 | 0 |
| Firmicutes | 0.2 | 0.05 | 1.98 | 0.04 | 0.2 | 0.15 | 0.27 | 0.19 | 0.08 | 15.89 | 4.89 | 3.99 | 1.44 | 0.09 |
| Fusobacteria | 0 | 0 | 0.7 | 0 | 0 | 0 | 0 | 0 | 0 | 3.19 | 0.12 | 0 | 0 | 0 |
| Gemmatimonadetes | 0 | 0 | 0 | 0 | 0 | 0 | 0 | 0 | 0 | 0.02 | 0.06 | 0 | 0 | 0 |
| Hydrogenedentes | 0.02 | 0.02 | 0.01 | 0 | 0 | 0.01 | 0 | 0 | 0.05 | 0 | 0 | 0 | 0 | 0 |
| Ignavibacteriae | 0.81 | 1.76 | 0.09 | 0.55 | 0.67 | 1.33 | 0.36 | 0.26 | 3.74 | 0 | 0.24 | 0.52 | 0.01 | 0 |
| Latescibacteria | 0 | 0.01 | 0.04 | 0 | 0 | 0 | 0 | 0 | 0 | 0 | 0.02 | 0 | 0 | 0 |
| Microgenomates | 0 | 0 | 0 | 0 | 0 | 0.11 | 0 | 0 | 0 | 0 | 0 | 0 | 0 | 0 |
| Nitrospirae | 0 | 0.1 | 0.05 | 0.24 | 0.01 | 0.36 | 0.19 | 3.18 | 1.08 | 0 | 0.06 | 5.01 | 0.53 | 6.03 |
| Omnitrophica | 0 | 0 | 0 | 0 | 0 | 0 | 0 | 0 | 0 | 0 | 0 | 0.01 | 0 | 0.03 |
| Parcubacteria | 0 | 0.02 | 0.02 | 0 | 0 | 0 | 0 | 0 | 0 | 0 | 0.06 | 0 | 0 | 0 |
| Planctomycetes | 0.29 | 3.04 | 1.15 | 2.24 | 0.87 | 1.3 | 0.5 | 0.09 | 0.56 | 3.14 | 3.78 | 0.17 | 0.23 | 0.14 |
| Proteobacteria | 44.69 | 54.13 | 47.93 | 49.14 | 23.42 | 24.49 | 47.45 | 7.76 | 42.39 | 60.32 | 27.5 | 21.54 | 8.06 | 1.63 |
| Spirochaetes | 0.12 | 0.11 | 0.27 | 0.06 | 0.22 | 0.75 | 0.96 | 0.19 | 1.43 | 0 | 0.14 | 0.22 | 0 | 0.12 |
| Thermodesulfobacteria | 0 | 0 | 0 | 0 | 0 | 0 | 0.02 | 0.74 | 0.01 | 0 | 0 | 0.13 | 0.02 | 0.15 |
| Thermotogae | 0 | 0 | 0 | 0 | 0 | 0 | 0.01 | 0.69 | 1.02 | 0 | 0 | 3.69 | 0.06 | 0.39 |
| Unclassified | 4.19 | 5.55 | 3.62 | 10.54 | 4.21 | 14.12 | 12.26 | 5.9 | 11.07 | 4.89 | 7.43 | 11.2 | 29.29 | 22.55 |
| Verrucomicrobia | 0.02 | 0.07 | 0.37 | 0.18 | 0.39 | 6.28 | 0 | 0.03 | 1.48 | 1.08 | 3.94 | 0.04 | 0.01 | 0 |
