# Supplementary material for: Temperature-controlled thermophilic bacterial communities in hot springs of western Sichuan, China
Source: BMC Microbiol. 2018 Oct 17;18:134. doi: 10.1186/s12866-018-1271-z (PMC6191902; doi:10.1186/s12866-018-1271-z)
Supplement: Supplementary file 5 — Table S4. The relative abundances (%) at genus level in different sample (DOCX 44 kb) [file 12866_2018_1271_MOESM5_ESM.docx]

**Additional file 5: Table S4. The relative abundances (%) at genus level in different sample**

|  | **ED** | **ZG-1** | **ZG-2** | **ZG-3** | **ZG-4** | **DB** | **MN** | **LL-1** | **LL-2** | **LL-3** | **LL-4** | **LL-5** | **LL-6** | **LL-7** |
| --- | --- | --- | --- | --- | --- | --- | --- | --- | --- | --- | --- | --- | --- | --- |
| Acidithiobacillus | 0.02 | 0.05 | 0 | 0.02 | 0 | 0 | 0 | 0.05 | 0.03 | 0 | 0.01 | 3.14 | 4.65 | 0 |
| Acinetobacter | 2.12 | 0.18 | 0.01 | 0.3 | 0.85 | 0.07 | 1.28 | 2.2 | 0.39 | 0.04 | 0.07 | 8.6 | 0.87 | 0.98 |
| Aeromonas | 0.19 | 0.01 | 0.01 | 0.07 | 0.04 | 0.35 | 0 | 0.01 | 0 | 1.73 | 0.14 | 0 | 0 | 0 |
| Algoriphagus | 0 | 0 | 0.1 | 0 | 0.05 | 0 | 0 | 0 | 0 | 0.16 | 0.01 | 0 | 0 | 0 |
| Alicyclobacillus | 0 | 0.01 | 0 | 0 | 0 | 0 | 0 | 0.01 | 0.01 | 0 | 0 | 0.59 | 0.99 | 0 |
| Alishewanella | 0 | 0.03 | 0 | 0.14 | 1.15 | 0.07 | 0 | 0 | 0.01 | 0 | 0 | 0 | 0 | 0 |
| Aquaspirillum | 0 | 0 | 0.16 | 0.02 | 0.01 | 0 | 0 | 0 | 0.01 | 0 | 6.16 | 0 | 0 | 0 |
| Armatimonas | 0.04 | 0 | 0 | 0 | 0 | 0 | 0 | 0 | 0 | 0.03 | 0.11 | 0 | 0 | 0 |
| Azospira | 0 | 0 | 8.82 | 0 | 0 | 0 | 0.06 | 0.01 | 0.13 | 0 | 0.67 | 0 | 0 | 0 |
| Bellilinea | 0 | 0.01 | 0 | 0.03 | 0 | 0.01 | 0.16 | 0.04 | 0.54 | 0 | 0 | 0.05 | 0.01 | 0 |
| Blastopirellula | 0.03 | 0.03 | 0.02 | 0 | 0 | 0 | 0 | 0 | 0.01 | 0.1 | 0.07 | 0 | 0 | 0 |
| Brevundimonas | 1 | 0.05 | 0 | 0 | 0 | 0 | 0 | 0.01 | 0.02 | 0.01 | 0.01 | 0 | 0 | 0 |
| Caldilinea | 0 | 0.71 | 0.02 | 1.01 | 0.03 | 0.04 | 0.08 | 0.44 | 0.2 | 0 | 0 | 0.04 | 0.01 | 0 |
| Caldimicrobium | 0 | 0 | 0 | 0 | 0 | 0 | 0.02 | 0.74 | 0.01 | 0 | 0 | 0.13 | 0.02 | 0.15 |
| Chitinibacter | 0 | 0 | 0 | 0.03 | 0 | 0 | 0 | 0 | 0 | 0 | 0.15 | 0 | 0 | 0 |
| Chitinophaga | 0.11 | 0.56 | 0.1 | 4.8 | 0.07 | 0.03 | 0 | 0.06 | 0.22 | 0.19 | 0.1 | 0.02 | 0.02 | 0 |
| Chloroflexus | 0.02 | 0.04 | 0 | 0.09 | 7.78 | 0.57 | 0.02 | 0.01 | 0.11 | 0 | 0 | 0 | 0 | 0.09 |
| Chryseobacterium | 0.03 | 0.05 | 0 | 0.04 | 0.01 | 0.33 | 11.23 | 0.01 | 0.05 | 0.01 | 0.04 | 0 | 0.01 | 0 |
| Clostridium | 0 | 0.01 | 0.03 | 0.01 | 0 | 0.01 | 0.01 | 0 | 0.01 | 14.58 | 0.37 | 0.01 | 0.01 | 0 |
| Dechloromonas | 0 | 0.05 | 7.85 | 0.05 | 0 | 0.02 | 0.08 | 0 | 0.15 | 0.01 | 0.7 | 0.09 | 0.01 | 0 |
| Dehalospirillum | 0 | 0 | 3.62 | 0 | 0 | 0 | 0.02 | 0 | 0.05 | 0.01 | 0 | 0 | 0 | 0 |
| Dictyoglomus | 0 | 0.01 | 0 | 0 | 0 | 0 | 0.01 | 1.97 | 0.02 | 0 | 0 | 0.49 | 0.02 | 1.34 |
| Duganella | 0 | 0 | 0 | 0 | 0 | 0.18 | 10.62 | 0 | 0.04 | 0 | 0.06 | 0.04 | 0 | 0 |
| Elioraea | 0 | 0.05 | 0 | 0.09 | 0.53 | 0 | 0.03 | 0.08 | 0.16 | 0 | 0 | 0.34 | 0.08 | 0.02 |
| Fervidobacterium | 0 | 0 | 0 | 0 | 0 | 0 | 0.01 | 0.69 | 1.02 | 0 | 0 | 3.69 | 0.06 | 0.39 |
| Flavobacterium | 0.2 | 0.04 | 2.71 | 0.15 | 0 | 0.29 | 11.91 | 0 | 0.04 | 0.03 | 0.05 | 0.01 | 0 | 0 |
| Fusibacter | 0 | 0 | 0.02 | 0 | 0 | 0 | 0 | 0 | 0 | 0 | 0.66 | 0 | 0 | 0 |
| Geminicoccus | 0 | 0.07 | 0 | 0.06 | 0.05 | 0.5 | 0.04 | 0.02 | 0.18 | 0.03 | 0.09 | 0.04 | 0.01 | 0.06 |
| Gemmata | 0.01 | 0.77 | 0.09 | 0.52 | 0.48 | 0 | 0.44 | 0.04 | 0.08 | 0.02 | 0.04 | 0.03 | 0.19 | 0.03 |
| Geobacter | 0 | 0.91 | 3.16 | 0.02 | 0.01 | 1.81 | 0.02 | 0 | 0.94 | 0 | 0.11 | 0 | 0.01 | 0 |
| Georgfuchsia | 0.04 | 0.07 | 0.01 | 0 | 0 | 0.94 | 0 | 0 | 0.02 | 0 | 0.11 | 0 | 0 | 0 |
| Hydrogenobacter | 0 | 0.14 | 0 | 0.05 | 0.18 | 0 | 1.97 | 56.82 | 0.32 | 0.01 | 0.02 | 5.53 | 29.02 | 2.96 |
| Hydrogenophaga | 0.84 | 5.09 | 0.41 | 1.55 | 1.92 | 0.28 | 0.36 | 0.29 | 1.22 | 0.21 | 0.14 | 0.12 | 0.07 | 0.01 |
| Hydrotalea | 0 | 0.01 | 0 | 0 | 0 | 0 | 0 | 0.01 | 0.02 | 0.01 | 0 | 2.69 | 0.06 | 0 |
| Hymenobacter | 0 | 0 | 0 | 0 | 0 | 0 | 0 | 0 | 0 | 0 | 0 | 0 | 0 | 0 |
| Ignavibacterium | 0.81 | 1.76 | 0.09 | 0.55 | 0.67 | 1.33 | 0.36 | 0.26 | 3.74 | 0 | 0.24 | 0.52 | 0.01 | 0 |
| Lewinella | 0.15 | 1.69 | 0.8 | 0.01 | 0 | 0.01 | 0 | 0.01 | 0.05 | 0.12 | 0.18 | 0 | 0.01 | 0 |
| Magnetospirillum | 0.01 | 0.11 | 0.35 | 0.02 | 0.04 | 0 | 0 | 0.01 | 0.01 | 0.01 | 1.16 | 0 | 0 | 0 |
| Massilia | 0.15 | 0.29 | 0.46 | 0.03 | 1.99 | 0.36 | 16.43 | 0.07 | 0.13 | 0.01 | 0.11 | 0.09 | 0.13 | 0.01 |
| Meiothermus | 0.01 | 0 | 0 | 0.4 | 2.56 | 0.02 | 0.08 | 0.6 | 2.69 | 0.09 | 0.11 | 1.98 | 0.2 | 0.05 |
| Methylocaldum | 0 | 0 | 0 | 0 | 0 | 0 | 0 | 0 | 0.59 | 0 | 0.01 | 0 | 0 | 0 |
| Methylococcus | 0 | 0.05 | 0.09 | 0 | 0 | 1.1 | 0 | 0 | 0.03 | 0 | 0.01 | 0 | 0 | 0 |
| Mycobacterium | 0 | 0.01 | 0 | 0 | 0 | 0 | 0 | 0 | 0.03 | 0 | 0 | 2.63 | 0.23 | 0 |
| Novispirillum | 0 | 0 | 0 | 0 | 0 | 0 | 0 | 0 | 0 | 0 | 0.7 | 0 | 0 | 0 |
| Novosphingobium | 0.79 | 6.82 | 0.23 | 1.19 | 0.75 | 0.25 | 0.1 | 0.35 | 1.06 | 0.14 | 0.28 | 0.13 | 0.05 | 0 |
| Ohtaekwangia | 0 | 0.26 | 0.33 | 0.16 | 0.12 | 0 | 0.01 | 0.01 | 0.02 | 0.43 | 0.88 | 0 | 0 | 0 |
| Opitutus | 0.01 | 0.04 | 0.26 | 0 | 0.03 | 0 | 0 | 0 | 0.01 | 0.47 | 2.17 | 0 | 0 | 0 |
| Paenibacillus | 0 | 0 | 0 | 0 | 0 | 0 | 0.05 | 0.01 | 0 | 0.03 | 0 | 1.14 | 0.01 | 0 |
| Paludibacter | 0.04 | 0 | 15.95 | 0 | 0 | 0 | 0.09 | 0 | 0.1 | 0.01 | 0.03 | 0.02 | 0.01 | 0 |
| Paracoccus | 1.67 | 0.07 | 0.03 | 0.03 | 0.04 | 0 | 0.01 | 0 | 0.01 | 0 | 0 | 0 | 0.01 | 0 |
| Pedobacter | 0 | 0.01 | 0 | 0 | 0 | 0.04 | 1.21 | 0.01 | 0 | 0.01 | 0.01 | 0 | 0.01 | 0.01 |
| Pelosinus | 0 | 0 | 0 | 0 | 0 | 0 | 0 | 0 | 0.01 | 0 | 0.01 | 0 | 0 | 0 |
| Planctomyces | 0 | 0.7 | 0.36 | 0.06 | 0.02 | 0 | 0 | 0 | 0.01 | 0.66 | 1.42 | 0 | 0 | 0 |
| Porphyrobacter | 1.48 | 0.51 | 0.1 | 0.18 | 0.24 | 0.02 | 0.05 | 0.01 | 0.06 | 0.13 | 0.11 | 0 | 0.01 | 0 |
| Propionivibrio | 0.02 | 0 | 0.59 | 0 | 0 | 0 | 0 | 0 | 0 | 0 | 0.14 | 0 | 0 | 0 |
| Pseudomonas | 0.09 | 0.2 | 0.08 | 12.08 | 4.3 | 0.45 | 16.4 | 0.47 | 0.14 | 51.38 | 0.14 | 0.44 | 0.08 | 0.01 |
| Psychrosinus | 0 | 0 | 0.25 | 0 | 0 | 0 | 0 | 0 | 0 | 0 | 0.01 | 0 | 0 | 0 |
| Rhizobium | 1.23 | 0 | 0.01 | 0.01 | 0 | 0 | 0.02 | 0.01 | 0.04 | 0 | 0.02 | 0 | 0 | 0 |
| Rhodobacter | 0.88 | 5.64 | 0.42 | 2.16 | 1.19 | 0.22 | 0.06 | 0.28 | 0.8 | 0.31 | 0.38 | 0.08 | 0.07 | 0 |
| Rickettsia | 0.01 | 3.26 | 0 | 0.01 | 0 | 0 | 0 | 0 | 0.01 | 0 | 0 | 0 | 0.05 | 0.01 |
| Roseiflexus | 0 | 0.02 | 0 | 0.89 | 0 | 0.77 | 1.12 | 0.04 | 0.32 | 0 | 0 | 0.07 | 0.04 | 0.27 |
| Rubribacterium | 0 | 0.01 | 0 | 0.84 | 0 | 0.01 | 0 | 0 | 0.02 | 0 | 0 | 0 | 0.01 | 0 |
| Ruminiclostridium | 0 | 0.01 | 0.69 | 0 | 0 | 0 | 0 | 0 | 0.02 | 0 | 0.25 | 0 | 0 | 0 |
| Sandaracinobacter | 1.13 | 0 | 0 | 0.17 | 0.01 | 0.01 | 0 | 0 | 0.04 | 0.15 | 0.15 | 0 | 0.01 | 0 |
| Silanimonas | 0 | 0 | 0.03 | 0 | 0.06 | 0.02 | 0 | 0.06 | 0.04 | 0.04 | 0.02 | 0.2 | 0.01 | 0 |
| Singulisphaera | 0 | 0.01 | 0 | 0 | 0 | 0 | 0 | 0 | 0 | 0 | 0 | 0 | 0 | 0 |
| Sphingomonas | 0.86 | 0.4 | 0.05 | 0.17 | 0.09 | 0.07 | 0.03 | 0.53 | 1.06 | 0.08 | 0.11 | 0.07 | 0.06 | 0 |
| Sphingopyxis | 0.04 | 0.02 | 0.13 | 0.03 | 0 | 0 | 0 | 0 | 0 | 0.13 | 0.71 | 0 | 0 | 0 |
| Spirosoma | 0.23 | 0 | 0 | 0 | 0 | 0 | 0 | 0 | 0 | 0 | 0 | 0 | 0 | 0 |
| Sulfurihydrogenibium | 0.12 | 0.39 | 0.03 | 0.63 | 1.82 | 0.04 | 0.35 | 7.76 | 0.41 | 0.05 | 0.04 | 27.25 | 6.33 | 51.85 |
| Sulfuritalea | 0 | 0 | 0.29 | 0.05 | 0 | 0.81 | 0 | 0 | 0 | 0 | 0.16 | 0 | 0 | 0 |
| Sulfurovum | 10.93 | 0.04 | 0 | 0.02 | 0 | 0 | 0 | 0.03 | 0.06 | 0.01 | 0 | 0 | 0.07 | 0 |
| Sunxiuqinia | 0 | 0.01 | 1.78 | 0 | 0 | 0 | 0 | 0 | 0 | 0 | 0.2 | 0 | 0 | 0 |
| Syntrophobacter | 0.01 | 0 | 0 | 0 | 0 | 0 | 0 | 0 | 0.65 | 0 | 0 | 0 | 0 | 0 |
| Tepidicella | 0 | 0.01 | 0 | 0.01 | 0.71 | 0.59 | 0 | 0.01 | 0.25 | 0 | 0.02 | 0.09 | 0 | 0 |
| Tepidimonas | 0.96 | 7.47 | 0.34 | 23.93 | 0.87 | 0.59 | 0.28 | 0.59 | 1.95 | 0.26 | 0.26 | 0.22 | 0.18 | 0.02 |
| Terriglobus | 0 | 0 | 0 | 0 | 0 | 0 | 0 | 0 | 0 | 0 | 0 | 0 | 0 | 0 |
| Thauera | 0 | 0 | 0 | 0.12 | 0.01 | 0 | 0 | 0 | 0 | 0 | 0 | 0 | 0 | 0 |
| Thermodesulfovibrio | 0 | 0.1 | 0 | 0.22 | 0.01 | 0.19 | 0.18 | 3.18 | 0.68 | 0 | 0.02 | 5.01 | 0.53 | 6.03 |
| Thermomonas | 0 | 0.01 | 0 | 0 | 0 | 0 | 0 | 0.06 | 0.01 | 0 | 0 | 1.71 | 0.01 | 0 |
| Thermus | 0 | 0.29 | 0 | 0.04 | 0.72 | 0.02 | 0.14 | 5.04 | 0.66 | 0.02 | 0.01 | 2.6 | 14.04 | 6.9 |
| Thiobacillus | 1.13 | 0.01 | 0.01 | 0 | 0.01 | 0.03 | 0 | 0 | 0.37 | 0.04 | 0.01 | 0 | 0 | 0 |
| Thiofaba | 1.93 | 0.01 | 0 | 0 | 0.22 | 1.99 | 0.01 | 0 | 0.43 | 0 | 0.01 | 0 | 0 | 0 |
| Thiothrix | 0.11 | 0.02 | 0 | 0 | 0.01 | 3.52 | 0 | 0.01 | 0.09 | 0.01 | 0.01 | 0 | 0 | 0 |
| Thiovirga | 3.69 | 0.01 | 0 | 0 | 0 | 0 | 0 | 0 | 0.01 | 0 | 0 | 0 | 0.01 | 0 |
| Tumebacillus | 0 | 0.01 | 0 | 0 | 0 | 0 | 0.01 | 0 | 0 | 0 | 0 | 1.13 | 0 | 0 |
| Undibacterium | 0.01 | 0.01 | 0.07 | 0 | 0 | 0 | 0 | 0.01 | 0.02 | 0 | 0.04 | 0.72 | 0.02 | 0 |
| Zavarzinella | 0.01 | 0.05 | 0.01 | 0.08 | 0.08 | 0.06 | 0 | 0 | 0.1 | 0.69 | 0.32 | 0 | 0.01 | 0 |
| Unclassified | 64.71 | 58.82 | 44.79 | 44.73 | 68.62 | 80.72 | 23.36 | 16.52 | 73.16 | 26.72 | 76.25 | 27.58 | 41.21 | 28.7 |
| Others (<0.5%) | 2.13 | 1.9 | 4.24 | 2.13 | 1.65 | 1.26 | 1.28 | 0.55 | 4.1 | 0.82 | 3.4 | 0.67 | 0.46 | 0.11 |
